# Determination of Complex Small‐Molecule Structures Using Molecular Alignment Simulation

**DOI:** 10.1002/anie.202000311

**Published:** 2020-02-24

**Authors:** Alain Ibáñez de Opakua, Frederik Klama, Ikenna E. Ndukwe, Gary E. Martin, R. Thomas Williamson, Markus Zweckstetter

**Affiliations:** ^1^ Structural Biology in Dementia German Center for Neurodegenerative Diseases (DZNE) Von-Siebold-Strasse 3a 37075 Göttingen Germany; ^2^ Department for NMR-based Structural Biology Max Planck Institute for Biophysical Chemistry Am Fassberg 11 37077 Göttingen Germany; ^3^ Analytical Research & Development (Rahway) Merck & Co. Inc. Kenilworth NJ USA; ^4^ Complex Carbohydrate Research Center University of Georgia Athens GA 30602 USA; ^5^ Department of Chemistry and Biochemistry Seton Hall University South Orange NJ 07079 USA; ^6^ Department of Chemistry & Biochemistry University of North Carolina Wilmington Wilmington NC 28409 USA

**Keywords:** configuration, NMR spectroscopy, stereochemistry

## Abstract

Correct structural assignment of small molecules and natural products is critical for drug discovery and organic chemistry. Anisotropy‐based NMR spectroscopy is a powerful tool for the structural assignment of organic molecules, but it relies on the utilization of a medium that disrupts the isotropic motion of molecules in organic solvents. Here, we establish a quantitative correlation between the atomic structure of the alignment medium, the molecular structure of the small molecule, and molecule‐specific anisotropic NMR parameters. The quantitative correlation uses an accurate three‐dimensional molecular alignment model that predicts residual dipolar couplings of small molecules aligned by poly(γ‐benzyl‐l‐glutamate). The technique facilitates reliable determination of the correct stereoisomer and enables unequivocal, rapid determination of complex molecular structures from extremely sparse NMR data.

## Graphical abstract

**A quantitative correlation** between the atomic structure of the alignment medium, the molecular structure of a small molecule, and molecule‐specific anisotropic NMR parameters has been established that predicts residual dipolar couplings of small molecules aligned by poly(γ‐benzyl‐l‐glutamate). These molecular alignment simulations allow determination of the relative configurations of small molecules from extremely sparse NMR data.

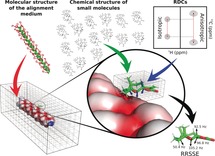

NMR spectroscopy is a powerful technique for assigning the constitution and configuration of small molecules and natural products. Insufficient NMR data or misinterpretation of available data, however, has led to structural misassignment of compounds,^1^ which in turn can lead to incorrect conclusions about structure–activity relationships or other important criteria. To overcome these problems, new methods based on anisotropic NMR parameters have been introduced.1b, 2 Anisotropic NMR‐based parameters are sensitive reporters of the global orientation of molecular bonds and chemical shielding tensors and provide a robust means for the structure assignment/validation of complex organic molecules.2a–2c, 3 Access to anisotropy‐based NMR parameters requires the generation of anisotropic environments in solution through the use of dedicated alignment media.2b, 3, 4 Despite intensive research,1b, 2b, 5 the connection between the structure of an alignment medium, the atomic structure of small molecules, and molecule‐specific anisotropy‐based NMR parameters has remained enigmatic.

Residual dipolar couplings (RDCs) provide a spatial view of the relative orientations of bonds, irrespective of the internuclear distances.2a, 2c, 4 For structure elucidation of small molecules and natural products, experimentally observed RDCs are compared with values expected on the basis of the proposed molecular constitution and configuration.1b, 2b In the absence of a connection between the atomic structure of the alignment medium and the expected RDCs, this comparison is based on a mathematical minimization procedure.2b Consequently, the utilization of RDCs for structural assignment strongly depends on the number and quality of experimentally observed RDCs, as well as the quality of the structure proposed for the small molecule.5e, 6 The technique, therefore, fails in cases where internuclear vectors are nearly parallel to each other or if they are located in a single plane. The measurement of RDCs in different alignment media may increase the amount of data available to determine the correct constitution and configuration.^7^ In addition, restraints based on residual chemical shift anisotropy (RCSA) minimize degeneracies when used in combination with RDCs, but RCSAs are more prone to measurement error than RDCs.2d, 8

Herein, we describe a molecular alignment simulation that accurately predicts RDCs in small molecules dissolved in organic solvents from the three‐dimensional structure of the alignment medium. The technique overcomes current limitations in the use of RDCs for the determination of complex molecular structures.

To establish a correlation between the structure of the alignment medium and RDCs in small molecules, we developed a structure‐based alignment model, termed P3D. P3D uses grid‐based sampling of solute positions/orientations in front of particles that form the alignment medium. Highly uniform grid‐based sampling overcomes the challenge of simulating very weak degrees of molecular alignment, which are required to retain the high resolution of the NMR spectra.^9^ Grid‐based alignment simulation has previously been shown to be very effective for predicting RDCs in biomolecules.^9^, ^10^ In contrast to this prior approach, which does not represent the alignment medium in atomic detail, the newly developed P3D simulation is based on the three‐dimensional structure of the alignment particle. In the P3D simulations, the small molecule is moved outward from the center of the alignment particle until all of its atoms are outside the van der Waals radius of all the atoms of the alignment particle. Then the alignment particle is sampled along the main axis, while its curved edge is sampled in angular steps, with the angle between two points remaining constant at increasing distances from the surface of the alignment particle. For each point on the grid and a predefined set of orientations, the potential energy between the alignment particle and the small molecule is calculated by solving the equations of continuum electrostatics.^11^ Potential energies are subsequently converted into probabilities for molecular orientations of the small molecule in front of the alignment particle using the Boltzmann equation. From these different weights for each of the points on the three‐dimensional grid, the alignment tensor is calculated, and from it the RDCs.

For implementation of P3D, we selected poly(γ‐benzyl‐l‐glutamate) (PBLG). PBLG has a well‐defined α‐helical structure and forms a lyotropic liquid‐crystalline phase, which is widely used for the measurement of RDCs in organic molecules.^12^ The three‐dimensional model of the PBLG particle was built by poling the α‐helical building block along its helical axis. Subsequently, the PBLG structure was energy‐minimized and equilibrated in a chloroform box using the molecular simulation software GROMACS.^13^

P3D was evaluated on the basis of RDCs measured for six small molecules dissolved in chloroform and weakly aligned by PBLG: the fungicidal cyclopentenone 4,6‐diacetylhygrophorone A (DAHPA),^14^ isopinocampheol (IPC),12a the alkaloids strychnine and caulamidine,^15^ 2‐(4‐chlorophenyl)‐5‐(dimethylphosphoryl)‐4‐phenylpyrrolidin‐3‐carboxylate (CPDMPPPC),^16^ and the lactone parthenolide.12b The six small molecules have different numbers of (relative) stereoisomers, ranging from 4 for DAHPA to 16 for parthenolide (see Figures S1–S6 in the Supporting Information). The 3D coordinates of the molecules and their stereoisomers were generated through computational chemistry methods on the basis of the corresponding constitution and configuration of each candidate.2b, 17 For all the molecules, a single enantiomeric form was present in the samples used for the RDC measurement. One‐bond CH RDCs are the largest RDCs in small molecules (Figure S7) and they can be measured with high accuracy.

The natural product DAHPA has two stereogenic centers in the cyclopentenone ring (C4 and C5; Figure S7) and one exocyclic center at C6, thus there are potentially eight stereoisomers.^14^ The absolute configuration of DAHPA is unknown. The four relative configurations 4*R*,5*S*,6*R*, 4*R*,5*R*,6*R*, 4*S*,5*R*,6*R*, and 4*R*,5*R*,6*S* are termed *RSR*, *RRR*, *SRR*, and *RRS* (Figures S1 and S7).

The PBLG‐induced alignment of the correct stereoisomer of DAHPA (*RSR*; reference^14^) was simulated using P3D. The axis corresponding to the largest eigenvalue of the P3D‐predicted alignment tensor (S_*zz*_) is oriented along the alkyl chain of DAHPA (Figure 1 a). Visualization of the alignment tensor axes in the context of a two‐dimensional world map shows that the orientation of the P3D‐predicted S_*zz*_ axis is highly similar to that derived by SVD‐based minimization (Figure 1 b, see also Figure S8). The angle between the P3D‐predicted and SVD‐derived S_*zz*_ axes is 2.5°. Good agreement is also observed between the simulated and SVD‐derived orientations of the other two alignment tensor axes (Figure 1 b, see also Figure S8). Since the S_*yy*_ and S_*xx*_ axes correspond to smaller eigenvalues (in this order), their orientation can show a larger spread. Comparison of the alignment tensors further demonstrated that the method used to generate the structure of the small molecule has little influence on the P3D‐predicted molecular alignment (Figure S9a).

**Figure 1:**
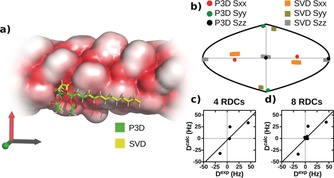
Molecular simulation predicts the PBLG‐induced alignment of a natural product. a) Visualization of the alignment of DAHPA in the anisotropic environment of a PBLG particle. The P3D‐predicted orientation is shown in the frame of the diagonalized alignment tensor (S_*zz*_, S_*yy*_, and S_*xx*_ axes shown in black, green, and red, respectively). The S_*zz*_ axis is parallel to the main axis of PBLG, which is oriented along the magnetic field. b) Comparison of the orientation of the P3D‐predicted alignment tensor (black, green, red) with the SVD‐derived orientation (gray, olive, orange; one‐bond and long‐range CH RDCs were used for SVD). The orientation of the three axes corresponding to the eigenvalues S_*zz*_, S_*yy*_, and S_*xx*_ of the diagonalized alignment tensor are projected onto a two‐dimensional world map. For the P3D prediction, the spread was derived from a 1 ns molecular dynamics simulation of PBLG (see Figure S10). For SVD, the spread was estimated using a Monte Carlo noise method.^6^ c, d) Correlation between RDCs predicted by P3D (*D* ^calc^) and experimental values (*D* ^exp^; (c), 4 one‐bond CH RDCs; (d), 4 one‐bond + 4 long‐range CH RDCs).

Next, the CH RDCs were calculated on the basis of the P3D‐predicted alignment tensor and compared to experimental values (Figure 1 c,d). Linear fitting of the *D* ^exp^ versus *D* ^calc^ representation resulted in a Pearson correlation coefficient (*R*) of 0.84 when considering only the four one‐bond CH RDCs (Figure 1 c). Inclusion of the small, long‐range CH RDCs did not affect the *R* value (Figure 1 d). Similar Pearson correlation coefficients were obtained when experimental RDCs were compared with those predicted for the structures generated by the cheminformatics software RDkit^18^ using either the Merck Molecular Force Field (MMFF) or the Universal Force Field (UFF; Figure S9b).

To evaluate the influence of changes in the atomic structure of PBLG on the P3D prediction, a 1 ns molecular dynamics simulation of a PBLG particle in a box of chloroform was performed. From the simulation, snapshots of the PBLG particle were taken every 10 ps (Figure S10a). For each snapshot, P3D simulations were performed for DAHPA, followed by a comparison of the predicted RDCs with the *D* ^exp^ values. Despite pronounced changes in the structure of the PBLG particle (Figure S10b), only small variations in the RDCs predicted for DAHPA (and the other five molecules), and thus the calculated Pearson correlation coefficient, were observed (Figure S10c). The analysis indicates that changes in the conformation of PBLG, such as bending of the PBLG particle and changes in side‐chain orientations, do not strongly affect the P3D simulations.

The complete relative configuration of DAHPA is difficult to establish using scalar couplings and NOEs, because of its quaternary carbon atom C5.^14^ Encouraged by the high quality of the P3D prediction for the *RSR* configuration (Figure 1), we performed P3D simulations on the other three stereoisomers (Figure 2). Linear fitting of the *D* ^exp^ versus *D* ^calc^ representation resulted in the Pearson correlation coefficients 0.73, 0.78, and 0.27 for the *RRR*, *SRR*, and *RRS* configurations, respectively. These *R* values are lower than that of the correct stereoisomer (*R*=0.84).

**Figure 2:**
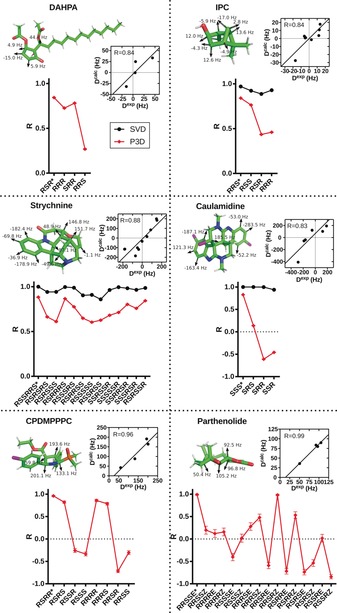
Comparison of P3D‐predicted RDCs (*D* ^calc^) with experimentally observed RDCs (*D* ^exp^) for different stereoisomers of the six small molecules (Figures S1–S7). The structure of the correct stereoisomer is shown together with experimental RDCs. *D* ^exp^ versus *D* ^calc^ representations of the correct stereoisomer are also shown. *R* values obtained by P3D simulations are displayed in red, those from SVD (when possible) in black. Correct stereoisomers are labeled with *. Error bars are calculated from 100 repetitions including noise in the RDCs.

The robustness and reliability of the P3D simulations were further evaluated for IPC, strychnine, caulamidine, CPDMPPPC, and parthenolide. IPC has three chiral centers and potentially eight stereoisomers (Figure S2). The four (relative) stereoisomers are 1*R*,2*R*,3*S*, 1*R*,2*S*,3*S*, 1*R*,2*S*,3*R*, and 1*R*,2*R*,3*R* subsequently termed *RRS*, *RSS*, *RSR*, and *RRR* (Figures S2 and S7). Linear fitting of the *D* ^exp^ versus *D* ^calc^ representation for the P3D prediction of the correct stereoisomer (*RRS*) resulted in a Pearson correlation coefficient of 0.84 (Figure 2). Smaller *R* values were obtained for the other three stereoisomers.

Ten one‐bond CH RDCs are available for determination of the relative configuration of strychnine.15a As a result of the quite large number of CH RDCs, the comparison of the *D* ^exp^ value with values best‐fitted to the structure using SVD can exclude many of the 13 possible stereoisomers (Figure 2).15a P3D prediction of the PBLG‐induced RDCs for the correct (*RSSRRS*) stereoisomer and comparison with the experimental RDCs resulted in a Pearson correlation coefficient of 0.88 (Figure 2). The P3D‐predicted RDCs for the other stereoisomers displayed lower agreement with *D* ^exp^ (with the exception of *RSRRRS*). Notably, the RDCs predicted for the correct stereoisomer by a previously developed 1D obstruction model^9^ correlated significantly worse with *D* ^exp^ (Figure S11), thus indicating that only P3D provides an accurate prediction of RDCs in small molecules aligned by PBLG.

In the case of caulamidine, only the RDCs predicted by P3D for the correct stereoisomer correlate with the experimental values (Figure 2), whereas the SVD‐based analysis gave *R* values close to 1.0 for all the stereoisomers (Figure 2).

The P3D‐predicted and experimental RDCs for the correct stereoisomer of CPDMPPPC were also very similar (*R*=0.96; Figure 2). The other relative stereoisomers of CPDMPPPC, in particular *RSSR*, *RSSS*, *RRSR*, and *RRSS*, have lower *R* values. Notably, only four one‐bond CH RDCs were reported for CPDMPPPC^16^—that is less than the minimum number required for SVD. Finally, the RDCs predicted by P3D for the correct stereoisomer (*RRSSE*) of parthenolide were nearly identical to experimental RDCs (*R*=0.99; Figure 2), while only one of the other 15 stereoisomers gave a *R* value above 0.6 (Figure 2).

To further improve the identification of the correct stereoisomer from P3D‐predicted RDCs, we define the quality parameter *RQ. RQ* is calculated as *(R*+1)^2^/*Q_s_*, where *Q_s_* is the RDC quality factor *Q*=rms(*D* ^exp^‐*D* ^calc^)/(rms *D* ^exp^), with rms=root‐mean square,8a scaled by the slope of the *D* ^exp^ versus *D* ^calc^ fitting. Scaling by the slope of the *D* ^exp^ versus *D* ^calc^ representation decreases inaccuracies in the P3D prediction of the alignment magnitude, which varies based on the concentration of the alignment medium. In addition, the use of *R*+1 in *RQ* avoids negative values, while the square enhances the relative importance of *R. RQ* approaches zero when the agreement between *D* ^exp^ and *D* ^calc^ is low. Notably, wrong stereoisomers can produce small *Q_s_* values and at the same time large negative *R* values when using P3D simulation (negative *R* values generally do not occur in SVD analysis). For the comparison of different stereoisomers, we further normalize the values to the largest *RQ* value observed for any of the possible stereoisomers. Calculation of the *RQ* values results in identification of the correct stereoisomer of all six molecules (Figure 3)—even when insufficient experimental RDCs are available to perform SVD.

**Figure 3:**
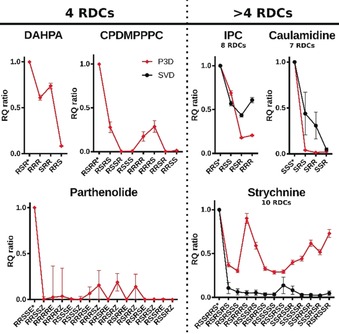
Normalized *RQ* ratios for P3D‐predicted RDCs (red) and SVD analysis (black, right). Correct stereoisomers are labeled with *. Error bars are calculated based on the *R* and *Q_S_* errors.

Inspection of the alignment tensor properties showed that the orientation of the S_*zz*_ axis of parthenolide was predicted with high accuracy (Figure S8). For the two other axes, the SVD‐derived orientation of the S_*yy*_ axis was closer to the P3D‐derived orientation of the S_*xx*_ axis and vice versa (Figure S8). This was also observed for strychnine and CPDMPPPC (Figure S8) and is related to the definition of the tensor parameters: the largest eigenvalue is labeled as S_*zz*_, followed by S_*yy*_, and then S_*xx*_. When S_*yy*_ and S_*xx*_ have similar magnitude, inaccuracies in the RDCs or alignment simulation can result in a “swap” of the S_*yy*_ and S_*xx*_ axes. This, however, has only a small influence on back‐calculated RDCs.

The P3D simulation uses the atomic structures of both the alignment medium and the solute, and calculates molecular interaction energies using a combination of steric obstruction and continuum electrostatics. Comparison with experimental NMR data showed that P3D predicts RDCs of small molecules aligned by PBLG with good accuracy (Figures 1–3). Despite very different chemical structures, the Pearson correlation coefficient between the predicted and experimental RDCs exceeds 0.82 for the correct stereoisomer of all the tested molecules and reaches 0.99 for parthenolide. The residual deviations between experimental and P3D‐predicted RDCs suggest that additional molecular interactions might be at work. In addition, inaccuracies in the electrostatic potential of PBLG and charges assigned to the small molecules might contribute. Considering the complexity of the problem, however, the high quality of the P3D prediction is remarkable.

Analysis of a large number of relative stereoisomers showed that P3D reliably identifies the correct configuration (Figures 2 and 3). The P3D‐based identification is based on similar criteria as SVD‐driven analysis: i) good agreement between experimental and calculated RDCs for at least one configuration, and ii) smaller *R* and/or RQ values for the other possible stereoisomers. The decision on what is a “good agreement” (i) and what is relative “smaller R or RQ” (ii) should take into account the different sources of inaccuracies, as investigated here: experimental measurement errors influencing *D* ^exp^ (in particular for long‐range RDCs), inaccuracies and dynamics in the structure of the small molecule, and, in the case of P3D, inaccuracies in the simulation. Supported by the data presented in Figures 2 and 3 we suggest that the Pearson correlation coefficient should exceed 0.8 for at least one of the possible stereoisomers as evidence for a good agreement between P3D simulation and experimental RDC data. When there are other stereoisomers with good agreement between *D* ^exp^ and *D* ^calc^, error estimates that consider all the above described inaccuracies have to be determined. In addition, both P3D and SVD analysis should be performed when a sufficient number of RDCs are available, because this increases the reliability of the identification of the correct relative configuration. Taking these considerations into account, the most striking application of P3D is probably the selection of the correct stereoisomer of parthenolide using only four CH RDCs from 16 possible relative configurations.

Alignment simulations might in the future also provide insight into the absolute configuration of small molecules.5a This will require the design of alignment media, which have i) a well‐defined molecular structure amenable to structural modeling, and ii) induce sufficiently divergent alignments of enantiomers. PBLG fulfills the first requirement, but the discriminative, RDC‐based power of PBLG for enantiomers is limited.12a, 19 An additional step might be the inclusion of specific interactions, such as salt bridges, into alignment simulations, and also the consideration of thermodynamic as well as kinetic contributions. Indeed, this step is tightly connected to the challenge of predicting the stereoselective affinity of the binding of small molecules to proteins.^20^

In summary, we established a quantitative connection between the alignment medium, the molecular structure of small molecules, and anisotropy‐based NMR parameters, and showed that this enables the determination of the relative configuration of small molecules from extremely sparse NMR data.

## Conflict of interest

The authors declare no conflict of interest.

## Supporting information

As a service to our authors and readers, this journal provides supporting information supplied by the authors. Such materials are peer reviewed and may be re‐organized for online delivery, but are not copy‐edited or typeset. Technical support issues arising from supporting information (other than missing files) should be addressed to the authors.

Supplementary
