# Supplementary material for: Determination of Complex Small‐Molecule Structures Using Molecular Alignment Simulation
Source: Angew Chem Int Ed Engl. 2020 Feb 24;59(15):6172–6. doi: 10.1002/anie.202000311 (PMC7187346; doi:10.1002/anie.202000311)
Supplement: Supplementary file 1 — Supplementary [file ANIE-59-6172-s001.pdf]

Angewandte Chemie
Eine Zeitschrift der Gesellschaft Deutscher Chemiker

Supporting Information

Determination of Complex Small-Molecule Structures Using Molecular Alignment Simulation

*Alain Ibáñez de Opakua, Frederik Klama, Ikenna E. Ndukwe, Gary E. Martin, R. Thomas Williamson, and Markus Zweckstetter**

anie_202000311_sm_miscellaneous_information.pdf

Supplementary Figures

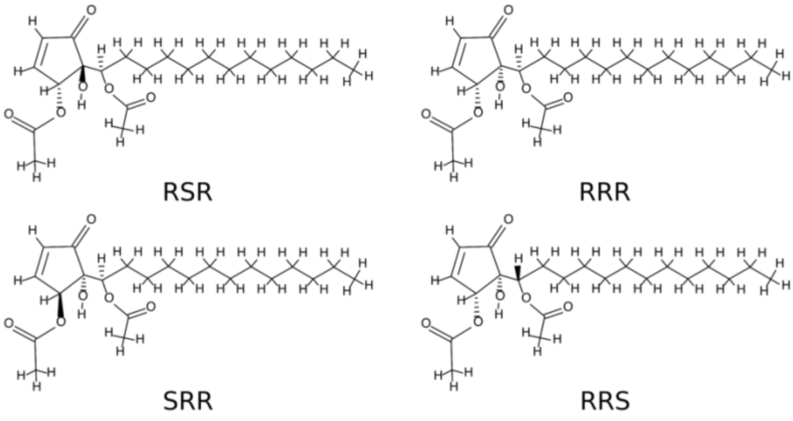

Figure S1. Stereoisomers of DAHPA. DAHPA has two stereogenic centers in the cyclopentenone ring (C4 and C5) and one exocyclic center at C6, thus potentially eight stereoisomers. The absolute configuration of DAHPA is unknown. The four relative configurations 4*R*,*5S*,*6R*, 4*R*,*5R*,*6R*, 4S,*5R*,*6R* and 4*R*,*5R*,*6S* are termed *RSR*, *RRR*, *SRR* and *RRS* (Fig. S7).

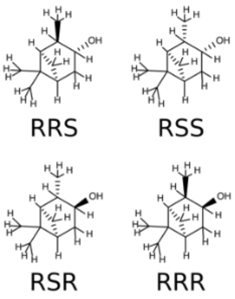

Figure S2. Stereoisomers of IPC. IPC has three chiral centers (C1 (alternatively C5), C2 and C3) and potentially eight stereoisomers. The levorotatory compound is used as reference (*1R*,*2R*,*3S*) and the other three stereoisomers are *1R*,*2S*,*3S*, *1R*,*2S*,*3R* and *1R*,*2R*,*3R* termed *RRS*, *RSS*, *RSR* and *RRR* (Fig. S7).

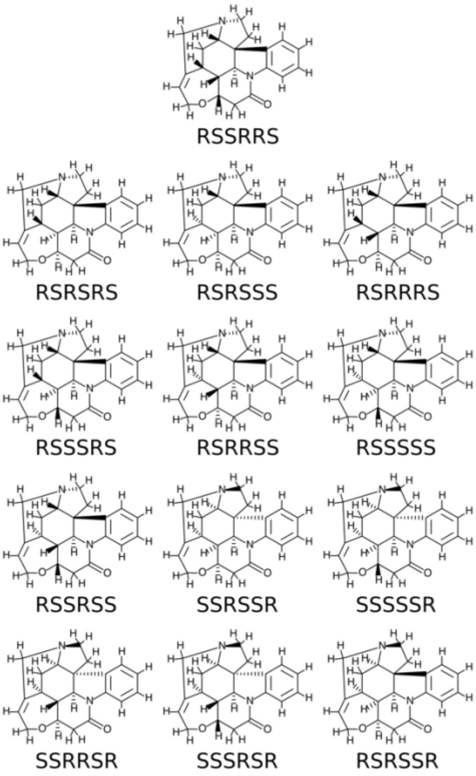

Figure S3. Stereoisomers of strychnine. Strychnine has six chiral centers (C7, C8, C12, C13, C14 and C16; Fig. S7) but from the expected 64 combinations only 26 are possible. The 13 relative stereoisomers generated are *7R,8S,12S,13R,14R,16S*, *7R,8S,12R,13S,14R,16S*, *7R,8S,12R,13S,14S,16S*, *7R,8S,12R,13R,14R,16S*, *7R,8S,12S,13S,14R,16S*, *7R,8S,12R,13R,14S,16S*, *7R,8S,12S,13S,14S,16S*, *7R,8S,12S,13R,14S,16S*, *7S,8S,12R,13S,14S,16R*, *7S,8S,12S,13S,14S,16R*, *7S,8S,12R,13R,14S,16R*, *7S,8S,12S,13R,14S,16R* and *7R,8S,12R,13S,14S,16R*, termed *RSSRRS, RSRSRS*, *RSRSSS, RSRRRS*, *RSSSRS*, *RSRRSS, RSSSSS, RSSRSS*, *SSRSSR*, *SSSSSR*, *SSRRSR*, *SSSRSR* and *RSRSSR* (Fig. S7).

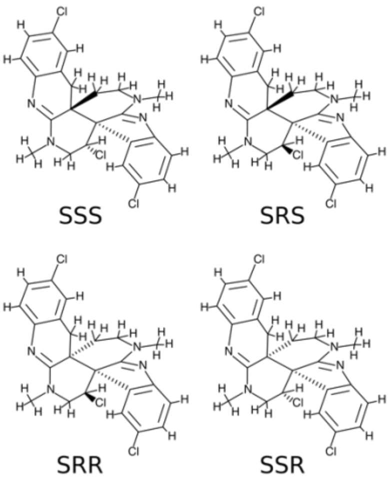

Figure S4. Stereoisomers of caulamidine. Caulamidine has three stereogenic centers in the heterocyclohexane ring (C10, C11 and C23) generating eight stereoisomers. The stereoisomers we use are *10S,11S,23S*, *10S,11R,23S*, *10S,11R,23R* and *10S,11S,23R*, termed *SSS*, *SRS*, *SRR* and *SSR* (Fig. S7).

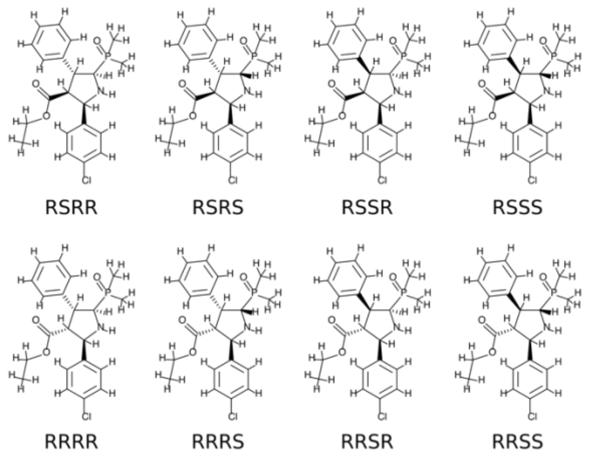

Figure S5. Stereoisomers of CPDMPPPC. CPDMPPPC has four chiral centers (C2, C3, C4 and C5) with 16 stereoisomers possible. The eight non-enantiomeric cases are *2R,3S,4R,5R*, *2R,3S,4R,5S*, *2R,3S,4S,5R*, *2R,3S,4S,5S*, *2R,3R,4R,5R*, *2R,3R,4R,5S*, *2R,3R,4S,5R* and *2R,3R,4S,5S*, termed *RSRR*, *RSRS*, *RSSR*, *RSSS*, *RRRR*, *RRRS*, *RRSR* and *RRSS* (Fig. S7).

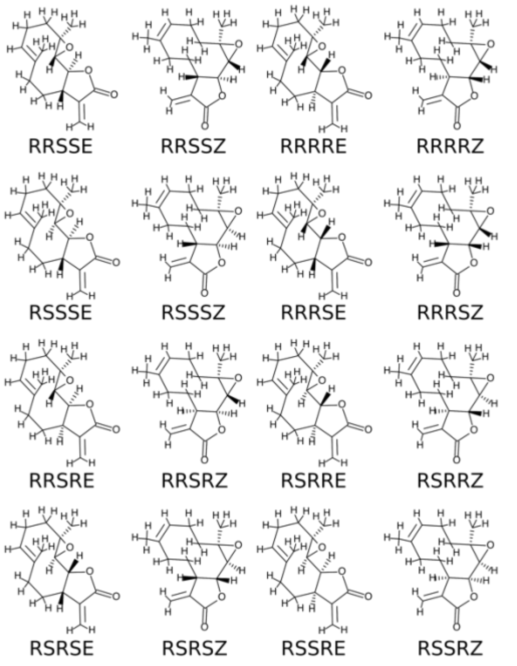

Figure S6. Stereoisomers of parthenolide. Parthenolide has four chiral centers (C3, C4, C7 and C8) and one geometric isomerism (between C5 and C6) generating 32 stereoisomers. The 16 relative configurations are *3R,4R,7S,8S,E*, *3R,4R,7S,8S,Z*, *3R,4R,7R,8R,E*, *3R,4R,7R,8R,Z*, *3R,4S,7S,8S,E*, *3R,4S,7S,8S,Z*, *3R,4R,7R,8S,E*, *3R,4R,7R,8S,Z*, *3R,4R,7S,8R,E*, *3R,4R,7S,8R,Z*,

3R,4S,7R,8R,E, *3R,4S,7R,8R,Z*, *3R,4S,7R,8S,E*, *3R,4S,7R,8S,Z*, *3R,4S,7S,8R,E* and *3R,4S,7S,8R,Z*, which are termed *RRSSE*, *RRSSZ*, *RRRRE*, *RRRRZ*, *RSSSE*, *RSSSZ*, *RRRSE*, *RRRSZ*, *RRSRE*, *RRSRZ*, *RSRRE*, *RSRRZ*, *RSRSE*, *RSRSZ*, *RSSRE* and *RSSRZ* (Fig. S7).

DAHPA

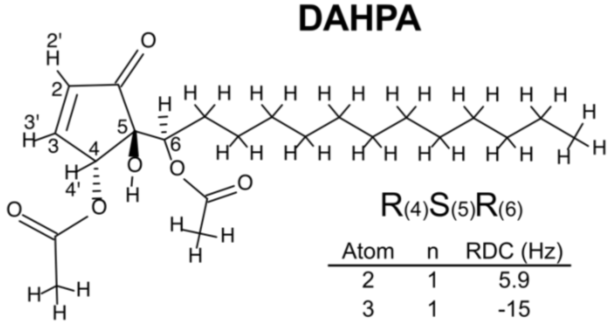

$R_{(4)}S_{(5)}R_{(6)}$

Atom	n	RDC (Hz)
2	1	5.9
3	1	-15
4	1	4.9
6	1	44.4
3-2'	1	-0.9
3-4'	1	4.1
4-2'	1	0.1
4-3'	1	0.5

Strychnine

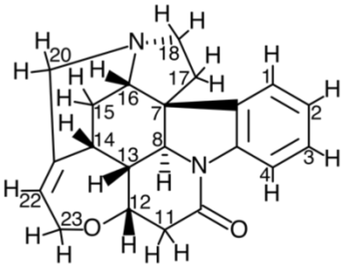

$R_{(7)}S_{(8)}S_{(12)}R_{(13)}R_{(14)}S_{(16)}$

Atom	n	RDC (Hz)
1	1	-178.9
2	1	-36.9
3	1	-69.8
4	1	-182.4
8	1	87.1
12	1	146.8
13	1	48.9
14	1	151.7
16	1	-49.6
22	1	-1.1

IPC

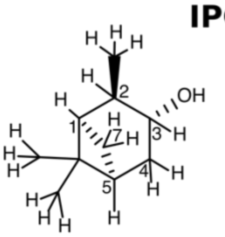

$R_{(1)}R_{(2)}S_{(3)}$

Atom	n	RDC (Hz)
1	1	13.6
2	1	-5.9
3	1	12
5	1	-4.9
4	2	-4.3<br>12.6
7	2	-17<br>2.8

Caulamidine

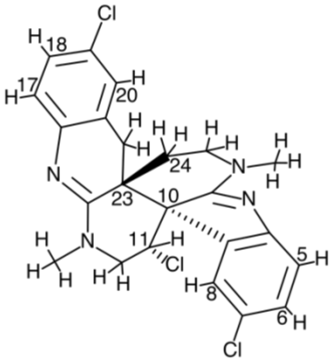

$S_{(10)}S_{(11)}S_{(23)}$

Atom	n	RDC (Hz)
5	1	-163.4
6	1	121.3
8	1	-187.1
11	1	185.5
17	1	-53
18	1	-283.5
20	1	-52.2

CPDMPPPC

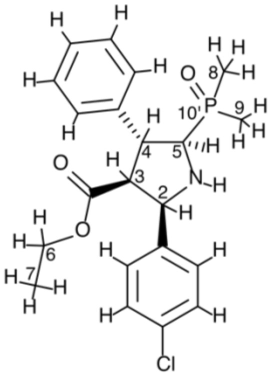

$R_{(2)}S_{(3)}R_{(4)}R_{(5)}$

Atom	n	RDC (Hz)
2	1	201.1
3	1	59.8
4	1	193.6
5	1	133.1
6-7	0	24.8
8-10'	0	-27.8
9-10'	0	-10

Parthenolide

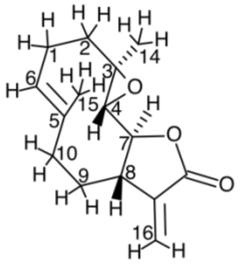

$R_{(3)}R_{(4)}S_{(7)}S_{(8)}E_{(5\text{-}6)}$

Atom	n	RDC (Hz)
4	1	105.2
6	1	50.4
7	1	92.5
8	1	96.8
3-14	0	-28.7
5-15	0	-14.7

Figure S7. Correct stereoisomers with experimental RDCs. In grey, additional RDCs needed for SVD-based analysis.

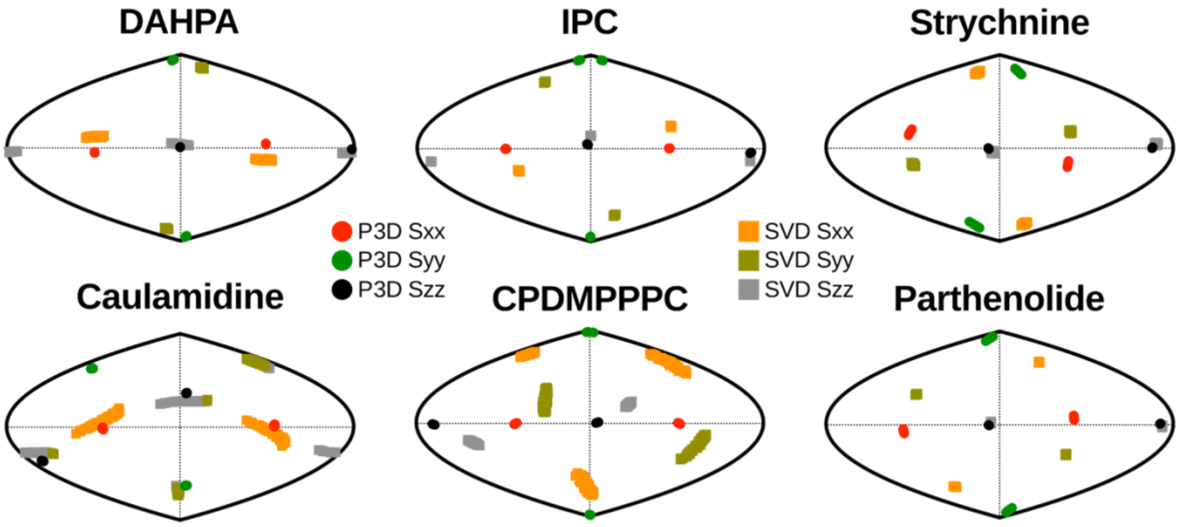

Figure S8. Visualization of the orientation of the alignment tensors predicted by P3D for the correct stereoisomers of the six small molecules with those derived by SVD. When less than five one-bond CH RDCs are available, the complete set of RDCs listed in Fig. S7 was used for SVD. In the case of strychnine, CPDMPPPC and parthenolide, the SVD-derived orientation of the S_{yy}-axis was closer to the P3D-derived orientation of the S_{xx}-axis and *vice versa*. This is related to the definition of the parameters of the alignment tensor, i.e. the largest eigenvalue is labelled as S_{zz}, followed by the second largest eigenvalue S_{yy}, and then S_{xx}. When S_{yy} and S_{xx} have similar magnitude, inaccuracies in RDCs or alignment simulation can result in a "swap" of the S_{yy}- and S_{xx}-axis, which however has only a small influence on the back-calculation of RDCs from the alignment tensor.

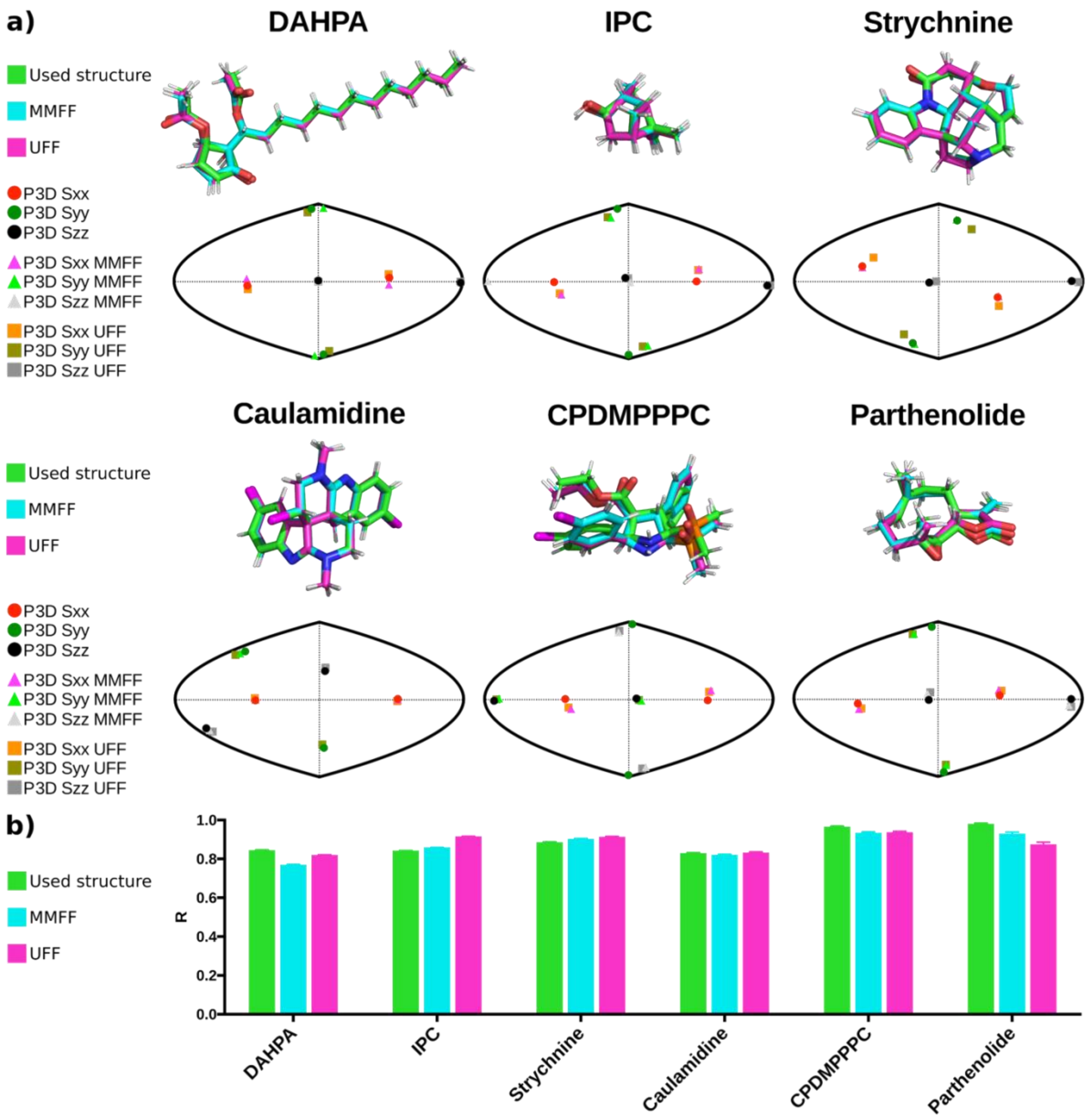

Figure S9. Influence of the molecule conformation on P3D prediction. **a)** For each small molecule the structure of the correct stereoisomer (green), which was used to obtain the results shown in Fig. 2-3, is compared to the structures optimized with the RDKit software using two different force fields, MMFF (cyan) and UFF (magenta). Below, the orientations of the alignment tensors obtained in each case are compared. **b)** Pearson correlation coefficients derived from D_{exp} *vs* D_{calc} representations. Colour scheme as in (a).

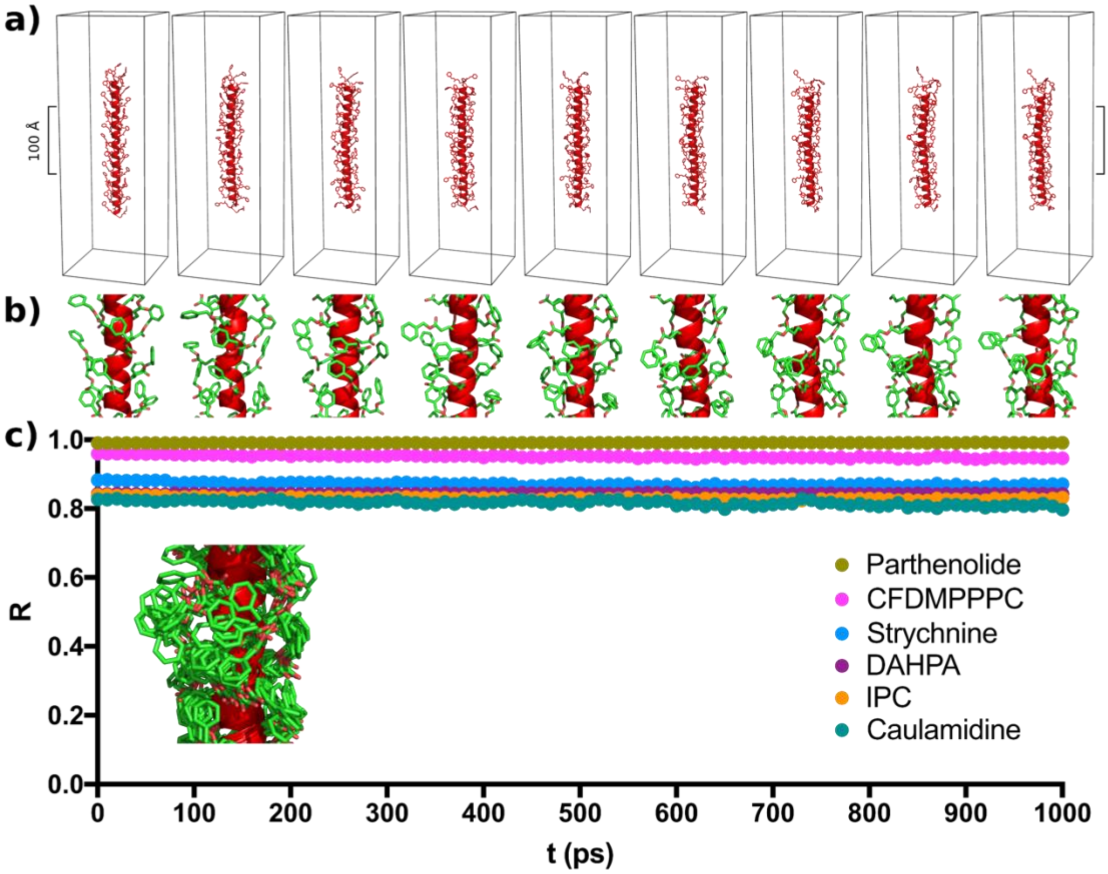

Figure S10. Influence of structural dynamics of PBLG on P3D prediction. **a-b)** Snap shots of the overall (a) and central part (b) of the PBLG structure along a 1 ns molecular dynamic simulation. **c,** Variation of D_{exp} *vs* D_{calc} Pearson correlation coefficients due to structural changes of the PBLG particle during the 1 ns molecular dynamic simulation of PBLG. The PBLG particle was aligned along the z-axis and one-bond CH RDCs were predicted by P3D using the 100 Å central part for each 10 ps snapshot and compared with the experimental RDCs of DAHPA, IPC, strychnine, caulamidine, CPDMPPPC and parthenolide. The superposition of all PBLG central part structures from (b) are shown inside the plot.

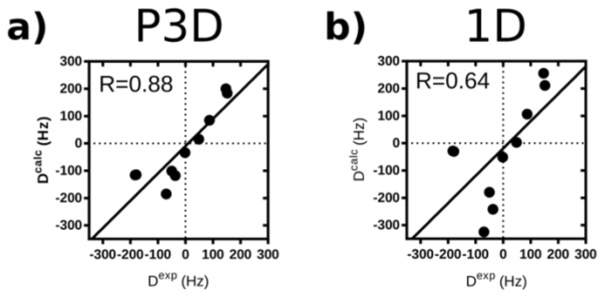

Figure S11. D_{exp} vs D_{calc} representations of RDCs predicted for strychnine by P3D (a, this work) and a previously developed 1D obstruction model (b)[1]. RDCs predicted by P3D correlate better with experimental RDCs (P3D: R = 0.88; 1D obstruction model: R = 0.64), indicating that only P3D provides accurate RDC prediction for small molecules aligned by PBLG.

Supplementary Methods

RDC measurement. Parthenolide was prepared as described previously.[2] To achieve an anisotropic environment, 20.5 mg of PBLG were added to a 170 µL $CDCl_3$ solution of parthenolide containing 3.5 mg of the sample. The sample was centrifuged back and forth in the tube multiple times to insure sample uniformity. Coupling constants were recorded using two-dimensional ^{13}C *J*-resolved NMR experiments,[3] acquired on a Bruker Avance III 500 MHz spectrometer equipped with a 5 mm TCI CryoProbeTM Prodigy. RDC values were measured as the difference between anisotropic and isotropic splittings. RDCs of the other molecules were reported previously.[3-4]

Conformer search and geometry optimization. Conformer search for parthenolide was performed with the Maestro Schrödinger package using its OPLS3e force field implementation,[5] which provides accurate geometries and adequate treatment of numerous functional groups and better represents lone electron pairs and charge distributions of drug-like molecules. Structures were kept in an energy window of 21 kJ/mol.

In the case of caulamidine, an in-house implementation of three conformer generation tools (ET[6], an in-house variant of distance geometry and OpenEye's OMEGA[7]) was used. For each molecule, the maximum number of generated conformations was set to 3000. All generated conformers were then clustered, after initial MMFF94 energy minimization,[8] based on rms of all atoms of 0.6 Å after superposition. Following a set of selection rules,[9] representative conformers were selected from the clusters. The conformers generated for caulamidine were subsequently re-optimized at a B3LYP/6-31G** level that uses the density functional theory–Hartree–Fock (HF) hybrid functional of B3LYP (Becke, three-parameter, Lee–Yang–Parr) and a split-valence double-zeta basis set 6-31G**, in which the core orbital is described by a single-basis function consisting of six Gaussian-type orbitals (GTOs) and each valence orbital is described by two basis functions (double-zeta)—one consisting of three GTOs, the other of one GTO.[10] The two asterisks in the basis set, sometimes also written as (*d*,*p*), indicate adding *d* polarization to non-hydrogen atoms and *p* polarization to hydrogens. Similarly, the conformers generated for parthenolide were re-optimized at M062X/6-31+G** level of theory, which uses the hybrid functional of Truhlar and Zhao with the split-valence double-zeta basis set but in this case including a diffuse function "+" (6-31+G**).

Strychnine structures were built as described previously.[11]

For DAHPA, IPC and CPDMPPPC, the structures were generated using the CORINA algorithm,[12] which combines monocentric fragments with standard bond lengths and angles. It handles rings and flexible chains separately in order to get an allowed set of torsion angles and minimize non-bonded interactions between flexible chain portions.

For the additional structures shown in Fig. S9, the RDKit software[13] was used together with the force fields MMFF and UFF.

Molecular alignment simulation. Prior to molecular alignment simulation, the structure of the PBLG particle was energy minimized and equilibrated in a chloroform box using GROMACS[14]. The chloroform solvent box was obtained from the list of equilibrated liquids validated for use with GROMACS and the OPLS/AA force field[15] at *virtualchemistry.org*.

The potential file of the PBLG particle was obtained through the Adaptive Poisson-Boltzmann Solver (APBS)[16] using the solvent dielectric constant of chloroform (4.8). Charges of the small molecules, for which RDCs were simulated, were calculated using *AtomicChargeCalculator* server[17] via the electronegativity equalization method based on a common charge calculation scheme (atoms-in-molecules) and a robust quantum mechanical approach (HF/6-311G).

The P3D simulation is based on a computationally fast method for deriving a molecular alignment tensor $\mathbf{A}_{\mathbf{mol}}$, which describes the average orientation of the solute molecule with respect to the magnetic field: the solute molecule is moved in steps on a three-dimensional grid that covers the central part of PBLG. At each step, an uniform distribution of different solute molecule orientations is sampled. The rotational sampling is achieved in a two-step process. First, the z-axis of solute molecule samples points, which were determined by a double cubic lattice method,[18] on a unit sphere. This sampling is highly uniform with the remaining deviation from completely uniform sampling corresponding to a residual alignment that is smaller than 10^{-7}.[1] With typical experimental alignment strength of ~10^{-3} this introduces negligible errors into the calculations. In a second step, the molecule is rotated around the *z*-axis in steps of 20°. For each orientation, the simulation evaluates whether the distance between any atom of the solute molecule and any atom of the PBLG particle is smaller than the sum of the two van der Waals radii. If this is not the case, an alignment matrix **A** is calculated according to A_{ij} = ½ (3 cos θ_i cos θ_j - δ_{ij}), where θ_i indicates the angle between the i_{th} molecular axis and the *z*-axis (magnetic field direction) and δ_{ij} the Kronecker delta. In addition, the interaction energy between the solute molecule and the PBLG particle is calculated for each orientation/grid position on the basis of the precomputed potential file of the PBLG particle and

the charges of the solute molecule. The interaction energy is converted into a Boltzmann weighing factor, which is multiplied to the alignment matrix **A**.[19] All rescaled alignment matrices are added together to obtain the molecular alignment tensor $\mathbf{A}_{\mathbf{mol}}$. The imperfect alignment of PBLG is taken into account by multiplication of $\mathbf{A}_{\mathbf{mol}}$ with the factor 0.8. In the final step, RDCs are calculated from $\mathbf{A}_{\mathbf{mol}}$ and compared to experimental values. The three-dimensional alignment model was implemented into the dipolar coupling analysis software PALES [20] using the C++ programming language.

We tested the convergence of the alignment simulation with respect to the spacing of the three-dimensional grid along which the geometric center of the molecule is moved. Increasing the spacing between grid points from 0.2 to 1.6 Å changes the orientation of the alignment tensor predicted for strychnine by 11.74°; so we selected a spacing of 0.4 Å, with only 1.26° difference. Similarly, sampling 500 instead of 100 orientations on the unit sphere and simultaneously increasing the sampling in the third dimension from 18 to 36, i.e., sampling a total of 18,000 instead of 1,800 orientations, changed the orientation of the alignment tensor predicted for strychnine by 0.61°. Therefore, 1,800 orientations were used for all results presented in this study.

Molecular dynamics simulation. Before the MD simulation, the PBLG model, prepared as described in the previous section, was equilibrated with 50,000 steps of initial energy minimization. To further equilibrate the system, 100 ps each of volume (NVT) and pressure (NPT) equilibration was performed. The MD simulation was carried out in a chloroform box using GROMACS (version 2018.2) along with the OPLS/AA force field at 300 K of temperature, 1 bar of pressure and with a coupling time (ζT) of 0.1 ps. The particle mesh Ewald algorithm was used for calculation of the electrostatic term, with a radius of 16 Å for the grid-spacing and Fast Fourier Transform. The cut-off algorithm was applied for the non-coulombic potential with a radius of 10 Å and LINCS algorithm was used to contain bonds and angles while carrying out the MD simulation. The simulation was performed during 1 ns in 2 fs steps and saving the coordinates of the system every 10 ps.

SVD of experimental RDCs. Experimental RDCs were best-fitted to the structures of the small molecules using SVD.[20] Variations in the SVD-derived quality measures *R* and *RQ* were evaluated using a Monte Carlo noise method,[21] in which random noise was added to the experimental RDCs according to their estimated accuracy (6.5 Hz for caulamidine, strychnine

and parthenolide; the noise applied to the RDCs of the other compounds was taken from the errors shown in the respective publications).

[1] M. Zweckstetter, A. Bax, *J Am Chem Soc* **2000**, *122*, 3791-3792.
[2] I. E. Ndukwe, X. Wang, I. Pelczer, M. Reibarkh, R. T. Williamson, Y. Liu, G. E. Martin, *Chem Commun (Camb)* **2019**, *55*, 4327-4330.
[3] Y. Liu, R. D. Cohen, G. E. Martin, R. T. Williamson, *J Magn Reson* **2018**, *291*, 63-72.
[4] aV. Schmidts, M. Fredersdorf, T. Lubken, A. Porzel, N. Arnold, L. Wessjohann, C. M. Thiele, *J Nat Prod* **2013**, *76*, 839-844; bA. Marx, V. Schmidts, C. M. Thiele, *Magn Reson Chem* **2009**, *47*, 734-740; cD. J. Milanowski, N. Oku, L. K. Cartner, H. R. Bokesch, R. T. Williamson, J. Sauri, Y. Liu, K. A. Blinov, Y. Ding, X. C. Li, D. Ferreira, L. A. Walker, S. Khan, M. T. Davies-Coleman, J. A. Kelley, J. B. McMahon, G. E. Martin, K. R. Gustafson, *Chem Sci* **2018**, *9*, 307-314; dJ. Becker, Karlsruher Instituts für Technologie (KIT) (Karlsruhe), **2018**.
[5] R. Berger, J. Courtieu, R. R. Gil, C. Griesinger, M. Kock, P. Lesot, B. Luy, D. Merlet, A. Navarro-Vazquez, M. Reggelin, U. M. Reinscheid, C. M. Thiele, M. Zweckstetter, *Angew Chem Int Ed Engl* **2012**, *51*, 8388-8391.
[6] B. P. Feuston, M. D. Miller, J. C. Culberson, R. B. Nachbar, S. K. Kearsley, *J Chem Inf Comput Sci* **2001**, *41*, 754-763.
[7] P. C. Hawkins, A. G. Skillman, G. L. Warren, B. A. Ellingson, M. T. Stahl, *J Chem Inf Model* **2010**, *50*, 572-584.
[8] T. A. Halgren, *J Comput Chem* **1996**, *17*, 490-519.
[9] E. C. Sherer, C. H. Lee, J. Shpungin, J. F. Cuff, C. Da, R. Ball, R. Bach, A. Crespo, X. Gong, C. J. Welch, *J Med Chem* **2014**, *57*, 477-494.
[10] Y. Liu, A. Navarro-Vazquez, R. R. Gil, C. Griesinger, G. E. Martin, R. T. Williamson, *Nat Protoc* **2019**, *14*, 217-247.
[11] G. Bifulco, R. Riccio, G. E. Martin, A. V. Buevich, R. T. Williamson, *Org Lett* **2013**, *15*, 654-657.
[12] .
[13] aN. O. Friedrich, C. de Bruyn Kops, F. Flachsenberg, K. Sommer, M. Rarey, J. Kirchmair, *J Chem Inf Model* **2017**, *57*, 2719-2728; b.
[14] S. Pronk, S. Pall, R. Schulz, P. Larsson, P. Bjelkmar, R. Apostolov, M. R. Shirts, J. C. Smith, P. M. Kasson, D. van der Spoel, B. Hess, E. Lindahl, *Bioinformatics* **2013**, *29*, 845-854.
[15] aW. L. Jorgensen, D. S. Maxwell, J. TiradoRives, *J Am Chem Soc* **1996**, *118*, 11225-11236; bG. A. Kaminski, R. A. Friesner, J. Tirado-Rives, W. L. Jorgensen, *J Phys Chem B* **2001**, *105*, 6474-6487.
[16] N. A. Baker, D. Sept, S. Joseph, M. J. Holst, J. A. McCammon, *Proc Natl Acad Sci U S A* **2001**, *98*, 10037-10041.
[17] C. M. Ionescu, D. Sehnal, F. L. Falginella, P. Pant, L. Pravda, T. Bouchal, R. Svobodova Varekova, S. Geidl, J. Koca, *J Cheminform* **2015**, *7*, 50.
[18] F. Eisenhaber, P. Argos, *J Comput Chem* **1993**, *14*, 1272-1280.
[19] M. Zweckstetter, G. Hummer, A. Bax, *Biophys J* **2004**, *86*, 3444-3460.
[20] M. Zweckstetter, *Nat Protoc* **2008**, *3*, 679-690.
[21] M. Zweckstetter, A. Bax, *J Biomol NMR* **2002**, *23*, 127-137.
